# Glutamatergic pathways in the brains of turtles: A comparative perspective among reptiles, birds, and mammals

**DOI:** 10.3389/fnana.2022.937504

**Published:** 2022-08-17

**Authors:** Mohammad Tufazzal Hussan, Akiko Sakai, Hideaki Matsui

**Affiliations:** ^1^Department of Neuroscience of Disease, Brain Research Institute, Niigata University, Niigata, Japan; ^2^Department of Anatomy and Histology, Patuakhali Science and Technology University, Barishal, Bangladesh

**Keywords:** glutamatergic neurons, vesicular glutamate transporters, glutamate receptors, reptiles, neurons, anoxia-tolerant, glutamate, excitotoxic cell death

## Abstract

Glutamate acts as the main excitatory neurotransmitter in the brain and plays a vital role in physiological and pathological neuronal functions. In mammals, glutamate can cause detrimental excitotoxic effects under anoxic conditions. In contrast, *Trachemys scripta*, a freshwater turtle, is one of the most anoxia-tolerant animals, being able to survive up to months without oxygen. Therefore, turtles have been investigated to assess the molecular mechanisms of neuroprotective strategies used by them in anoxic conditions, such as maintaining low levels of glutamate, increasing adenosine and GABA, upregulating heat shock proteins, and downregulating K_*ATP*_ channels. These mechanisms of anoxia tolerance of the turtle brain may be applied to finding therapeutics for human glutamatergic neurological disorders such as brain injury or cerebral stroke due to ischemia. Despite the importance of glutamate as a neurotransmitter and of the turtle as an ideal research model, the glutamatergic circuits in the turtle brain remain less described whereas they have been well studied in mammalian and avian brains. In reptiles, particularly in the turtle brain, glutamatergic neurons have been identified by examining the expression of vesicular glutamate transporters (VGLUTs). In certain areas of the brain, some ionotropic glutamate receptors (GluRs) have been immunohistochemically studied, implying that there are glutamatergic target areas. Based on the expression patterns of these glutamate-related molecules and fiber connection data of the turtle brain that is available in the literature, many candidate glutamatergic circuits could be clarified, such as the olfactory circuit, hippocampal–septal pathway, corticostriatal pathway, visual pathway, auditory pathway, and granule cell–Purkinje cell pathway. This review summarizes the probable glutamatergic pathways and the distribution of glutamatergic neurons in the pallium of the turtle brain and compares them with those of avian and mammalian brains. The integrated knowledge of glutamatergic pathways serves as the fundamental basis for further functional studies in the turtle brain, which would provide insights on physiological and pathological mechanisms of glutamate regulation as well as neural circuits in different species.

## Introduction

Glutamate, the principal excitatory neurotransmitter in the vertebrate brain, is involved in normal brain functions such as learning and memory. In spite of its essential roles, glutamate also plays a detrimental role in the excitotoxic cell death in neurologic disorders such as cerebral stroke, brain injury, Alzheimer’s disease, Parkinson’s disease, Huntington’s disease, and epilepsy (Liguz-Lecznar and Skangiel-Kramska, 2007). For studying the molecular mechanisms of neuroprotective pathways in anoxic conditions that induce excitotoxicity in mammalian brains, turtles are becoming an ideal animal model because turtles can endure a prolonged anoxic environment and postanoxic reoxygenation without brain damage (Milton and Prentice, 2007; Milton, 2019). Mammalian brains are considerably sensitive to oxygen deprivation. Even a small shortage of oxygen can cause a decrease in adenosine triphosphate (ATP) levels in the cell (Kristian, 2004). Consequently, disturbance of membrane potentials occurs due to malfunction of the ion pumps which constantly require ATP, triggering the release of excitatory amino acid neurotransmitters such as glutamate (Kirdajova et al., 2020). The glutamate then binds to the glutamate receptors present in the postsynaptic membrane, such as the α-amino-3-hydroxy-5-methyl-4-isoxazole propionic acid (AMPA) receptor and the *N*-methyl-D-aspartate (NMDA) receptor, which are ligand-activated ion channels (Blanke and VanDongen, 2009; Zanetti et al., 2021). The stimulation of glutamate receptors enhances the entry of calcium (Ca^2+^) into the cell, which activates proteases, lipases, and endonucleases. These enzymes then destroy cellular integrity, which results in excitotoxic cell death (Kritis et al., 2015; Jones, 2020; Kirdajova et al., 2020). By contrast, the freshwater turtle *Trachemys scripta* can tolerate days of anoxia at room temperature for months without oxygen at 3°C (Milton, 2019). The protective pathways in the turtle brain are similar to those in the mammalian brain observed by ischemic/hypoxic preconditioning. In both cases, the extracellular glutamate levels remain low (Milton et al., 2002; Thompson et al., 2007). However, considering the susceptibility of mammalian brain cells to hypoxia, it may be difficult to differentiate between adaptive and injurious responses. We speculate that distinct and/or common molecular pathways participate in both cases. Thus, critical protective pathways could possibly be identified by using the turtle as a constitutively anoxic preconditioned model (Milton and Prentice, 2007; Milton, 2019; Wijenayake and Storey, 2021).

In the brain of the turtle, glutamate was demonstrated to be an excitatory neurotransmitter by pharmacological experiments in which neurons stopped firing after the injection of ionotropic glutamate receptor antagonists (Larson-Prior et al., 1991, 1995; Berkowicz et al., 1994; Broglio et al., 2015). Pharmacological research in the medial cortex of the turtle also showed that glutamate is involved in learning and memory (Broglio et al., 2015). Moreover, intriguingly, glutamate appears to control ion channels in turtles as a strategy to survive in oxygen-deprived situations (Pamenter et al., 2008b; Couturier et al., 2019). Thus, glutamate is an important neurotransmitter in the brains of turtles, and using turtles as a research model is beneficial. However, the glutamate circuitry of the turtle brain is still not fully demonstrated. The messenger ribonucleic acid (mRNA) of vesicular glutamate transporters (VGLUTs) has been found in the soma of glutamatergic neurons (Muñoz et al., 1999; Fremeau et al., 2001; Islam and Atoji, 2008); thus, its expression could be used as a proxy for glutamatergic pathways. Conversely, mRNA of glutamate receptors (GluRs) is expressed by the neurons receiving glutamatergic afferents, and their expression could be marked as the projection targets of glutamatergic neurons (Conti et al., 1994; Muñoz et al., 1999; Fremeau et al., 2001; Islam and Atoji, 2008; Karim et al., 2014). In this review, our first goal is to discuss the candidate glutamatergic pathways in turtle brains based on the origin and projection targets of glutamatergic neurons along with hodological data. Our second goal is to compare the distribution of glutamatergic neurons in the pallium of reptiles, birds, and mammals. Developmental data on transcription factors that play critical roles in cortical development in mammals (Pax6, Emx1/2, and Tbr1) show a conserved expression pattern on the dorsal side of the amniote telencephalon, implying that all amniotes have a homologous brain part in the telencephalon, termed the pallium (Fernandez et al., 1998; Puelles et al., 2000, 2017; Dugas-Ford et al., 2012; Desfilis et al., 2017; Medina et al., 2021). Using mouse embryos, Emx1- and Tbr1-expressing neurons in the dorsal part of the telencephalon (pallium) were shown to use glutamate as a neurotransmitter (Gorski et al., 2002; Hevner et al., 2003, 2006). However, the adult avian and reptilian brains lack the mammalian-like pallial region. Nonetheless, pallial structures in the adult avian and reptilian brains still conserve several characteristics of the mammalian pallium, such as neurons in the pallium are predominantly excitatory and less inhibitory (Medina and Reiner, 2000; Medina, 2007; Suryanarayana et al., 2017; Spool et al., 2021; Ditz et al., 2022). The principal pallial neurons are glutamatergic, as demonstrated by the examination of the expression pattern of VGLUT1 and VGLUT2 in adult mammalian brains (Ni et al., 1995; Fremeau et al., 2001; Broman et al., 2004) and by the localization of VGLUT2 in adult avian brains (Islam and Atoji, 2008; Karim et al., 2014). The neocortex of the mammalian brain has six layers, and the reptilian cortex has three layers. The reptilian brains have an additional structure named the dorsal ventricular ridge (DVR), which is absent in mammals. On the other hand, the avian brains have a DVR but they do not have a mammalian-like layered cortex (Lohman and Smeets, 1990; Butler, 1994). Therefore, we also intended to focus on the pallial organization of glutamatergic neurons among amniotes.

**FIGURE 1:**
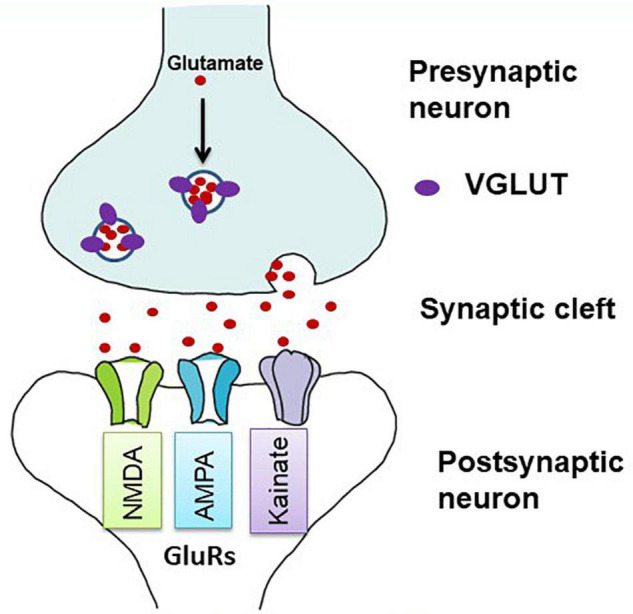
Diagrammatic illustration of glutamatergic neurotransmission in the synapse. Vesicular glutamate transporters (VGLUTs) transport glutamate into synaptic vesicles at presynaptic terminals. In the synaptic cleft, glutamate is released by exocytosis and stimulates glutamate receptors (GluRs) on postsynaptic membranes (adapted from Kanai and Hediger, 2004). Both ionotropic and metabotropic receptors are responsible for glutamatergic neurotransmission. Here, only ionotropic receptors are shown as they act as ion channels and are mostly involved in the excitotoxic cell death.

Glutamate is the principal excitatory neurotransmitter in the brain, and its transmission is primarily regulated by VGLUTs and GluRs (Figure 1). In addition to physiological roles in the brain, such as learning and memory, glutamate has a detrimental effect on excitotoxic cell death in the ischemic cascades associated with cerebral stroke and neurodegenerative diseases (Jones, 2020). Upon nerve stimulation, VGLUTs transport glutamate through synaptic vesicles at the presynaptic end of the axon, and glutamate is released from the vesicles to the synaptic cleft. When this glutamate binds to GluRs at the postsynaptic terminal (Figure 1), neural excitation is transmitted (Groc and Choquet, 2020). Three isoforms of VGLUTs (VGLUT1, VGLUT2, and VGLUT3) have been identified and extensively studied in mammals (Table 1; Ni et al., 1994; Aihara et al., 2000; Gras et al., 2002; Herzog et al., 2004). To identify glutamatergic neurons, VGLUT1 and VGLUT2 are used as biomarkers in mammals, and their distribution in the brain is complementary (Ni et al., 1994, 1995; Hisano et al., 2000; Fremeau et al., 2004). The prominent expression of VGLUT1 mRNA is found mainly in the cerebral cortex, the hippocampus, and the cerebellar cortex, whereas VGLUT2 mRNA is strongly expressed in the amygdaloid nuclei, thalamus, hypothalamus, lower brainstem, and cerebellar nuclei. On the other hand, VGLUT3 mRNA is detected in other types of neurons that use gamma-aminobutyric acid (GABA), acetylcholine, and serotonin as their neurotransmitters and in astrocytes (Bai et al., 2001; Gras et al., 2002; Kawano et al., 2006). In birds, the VGLUT2 and VGLUT3 genes have been identified, and their distribution in the brain has been studied (Table 1; Islam and Atoji, 2008; Atoji, 2011; Atoji and Karim, 2014a; Karim et al., 2014; Colquitt et al., 2021). Avian VGLUT1 has not been found yet and avian VGLUT2 bears characteristics of both VGLUT1 and VGLUT2 of mammals regarding its distribution in the brain. Thus, there is a possibility that avian VGLUT1 has been lost during evolution (Atoji, 2011). VGLUT2 mRNA is found strongly in the pallium, thalamus, midbrain, brainstem, and cerebellar cortex of the avian brain. Glutamatergic neurons labeled with avian VGLUT2 mRNA and protein are located in regions corresponding to those in which VGLUT1 or VGLUT2 are expressed in the mammalian brain. Avian VGLUT3 mRNA is expressed only in a serotonergic nucleus called the caudal linear nucleus (Atoji and Karim, 2014a). Furthermore, the distribution of GluRs, such as AMPA, kainite, and NMDA receptors, has been extensively studied in mammalian (Keinanen et al., 1990; Sato et al., 1993; Conti et al., 1994) and avian brains (Ottiger et al., 1995; Wada et al., 2004; Islam and Atoji, 2008; Karim et al., 2014).

**TABLE 1:** Regional distribution of vesicular glutamate transporter (VGLUTs) mRNA in the brain of the reptiles, birds, and mammals.

		Reptiles			Birds			Mammals			
	Brain regions	VGLUT1	VGLUT2	VGLUT3	Brain regions	VGLUT2	VGLUT3	Brain regions	VGLUT1	VGLUT2	VGLUT3
**(A)**	**Telencephalon**										
1	Mitral cells of olfactory bulb	+++	++	−	Mitral cells of olfactory bulb	+++	−	Mitral cells of olfactory bulb	+++	+	−
2	Lateral cortex	+++	+	−	Hyperpallium	++	−	Cortical layer I	+	−	−
3	Dorsal cortex	+++	+	−	Mesopallium	+++	−	Cortical layer II	+++	−	++
4	Dorsomedial cortex	+++	+	−	Nidopallium	++	−	Cortical layer III	+++	+	+
5	Medial cortex	+++	+	−	Hippocampal formation	++	−	Cortical layer IV	+++	+	+
6	Dorsal ventricular ridge (DVR)	+++	+	−	Arcopallium	++	−	Cortical layer V	+++	+	+
7	Medial septal n.	−	+	−	N. teniae of amygdala	++	−	Cortical layer VI	+++	−	+
8	Lateral amygdaloid n.	+	+	−	Posterior pallial amygdala	+++	−	Hippocampus	+++	+	+
9	Medial amygdaloid n.	++	+	−	Dorsolateral corticoid area	++	−	Lateral amygdala	+	−	+
10	Lateral olfactory tract n.	+	−	−	Piriform cortex	++	−	Caudate−putamen	−	−	+
**(B)**	**Diencephalon**										
1	Lateral habenular n.	−	+	−	Lateral habenular n.	++	−	Lateral habenular n.	−	+++	−
2	Medial habenular n.	−	++	−	Medial habenular n.	+++	−	Medial habenular n.	++	++	−
3	Dorsomedial thalamic n.	−	++	−	Dorsolateral anterior thalamic n.	++	−	Subthalamic n.	−	+++	−
4	Dorsolateral thalamic n.	−	+++	−	Dorsomedial anterior thalamic n.	++	−	Dorsal lateral geniculate n.	+	++	−
5	Dorsolateral geniculate n.	−	+	−	Dorsomedial posterior thalamic n.	++	−	Medial geniculate body	−	++	−
6	N. reuniens	−	+	−	Dorsointermedial posterior thalamic n.	++	−	Ventral posterolateral n.	++	++	−
7	Rotundal n.	−	+	−	Dorsolateral posterior thalamic n.	+++	−	Ventral posteromedial n.	++	++	−
8	Periventricular hypothalamic n.	−	++	−	Superficial parvocellular n.	+++	−	Posterior complex	−	++	−
9	Lateral hypothalamic area	−	+	−	Lateral anterior thalamic n.	++	−	Ventral anterior−lateral complex	++	++	−
10					Ventral lateral geniculate n.	++	−	Ventral medial n. (VM)	−	++	−
11					Ovoidal n.	+++	−	Mediodorsal n.	++	++	−
12					Triangular n.	+++	−	Submedial n.	++	+	−
13					Rotundal n.	+++	−	Anterodorsal n.	++	+++	−
14					Subrotundal n.	+++	−	Anteromedial n.	−	+++	−
15					Hypothalamic area	+	−	Anteroventral n.	+	++	−
16								Lateral posterior n.	+	++	−
17								Lateral dorsal n.	++	++	−
18								Parataenial n.	−	++	−
19								Interanteromedial n.	−	+++	−
20								Intermediodorsal n.	−	+++	−
21								N. reuniens	−	+++	−
22								Rhomboid n.	−	+++	−
23								Central medial n.	−	+++	−
24								Paracentral n.	−	+++	−
25								Central lateral n.	−	+++	−
26								Parafascicular n.	−	+++	−
27								Hypothalamic area	−	+/++	−
**(C)**	**Mesencephalon**										
1	Periventricular cellular layer	+	+	−	Tectal layer 13	+++	−	Stratum griseum superficiale of SC	−	+++	−
2	Torus semicircular central n.	−	+	−	Tectal layers 8,15	++	−	Stratum opticum of SC	−	+	−
3	Magnocellular part of isthmic n.	−	+++	−	Tectal layers 2,4,6,9−12,14	+	−	Stratum griseum intermediale of SC	−	+	−
4	Parvocellular part of isthmic n.	−	−	++	Magnocellular part of isthmic n.	−	−	Stratum album intermediale of SC	−	+	−
5					Parvocellular part of isthmic n.	+++	−	Stratum griseum profundum of SC	−	+	−
6					Lateral mesencephalic n., dorsal part	+++	−	Stratum album profundum of SC	−	−	−
7					Intercollicular n.	++	−	Periaqueductal gray of SC	−	+++	−
8					Isthmo−optic n.	++	−	Inferior Colliculus	−	+++	−
9					Ruber n.	++	−				
**(D)**	**Rhombencephalon**										
1	Granular layer of cerebellar cortex	+++	−	−	Granular layer of cerebellar cortex	+++	−	Granular layer of cerebellar cortex	+++	−	−
2	Cochlear n.	+	++	+	Cerebellar n.	++	−	Cerebellar n.	−	+++	−
3	Tangential vestibular n.	+	++	−	Lateral lemnisci n., ventral part	++	−	Intermediate n. of lateral lemniscus	+	+++	−
4	Ventrolateral vestibular n.	+	++	−	Lateral lemnisci n., dorsal part	++	−	Lateral reticular n.	++	+++	−
5	Descending n. of the trigeminus	+	+	−	Principal sensory n. of trigeminal nerve	+++	−	External cuneate n.	+	+++	−
6	Lateral cerebellar n.	−	++	−	Pontine n.	+++	−	Inferior olivary n.	−	+++	++
7	Medial cerebellar n.	−	+++	−	Superior olivary n.	+	−	Spinal trigeminal n.	+	+++	+
8	Superior vestibular n.	−	+	−	Inferior olivary n.	+++	−	Gracile n.	+	+++	−
9	Superior reticular n.	−	+++	−	Vestibular n.	++	−	Cuneate n.	+	+++	−
10	Principal sensory n. of trigeminal nerve	−	+	−	Cochlear n.	++	−	Solitary tract n.	−	+++	+
11	Superior raphe n.	−	−	+++	Reticular n.	++	−	Vestibular n. complex	+	++	−
12	Inferior raphe n.	−	−	+	Caudal linear n.		++	Cochlear n.	+++	++	−
13	Median reticular n.	+	+	−			−	Pontine n.	+++	+	−
14								Raphe n.	++	+++	++
15								Superior olivary n.	+	++	−
**(E)**	**References**										
	Hussan, 2016; Hussan et al., 2017; Sarkar and Atoji, 2018				Islam and Atoji, 2008; Atoji and Karim, 2014a; Karim et al., 2014			Ni et al., 1995; Hisano et al., 2000, 2002; Fremeau et al., 2001, 2002, 2004; Gras et al., 2002; Schafer et al., 2002; Barroso-Chinea et al., 2007; Balaram et al., 2011; Ito et al., 2011; Vigneault et al., 2015			

+++, intense expression; ++, moderate expression; +, weak expression; −, no expression; VGLUT, vesicular glutamate transporter; SC, superior colliculus; N., nucleus/nuclei.

**FIGURE 2:**
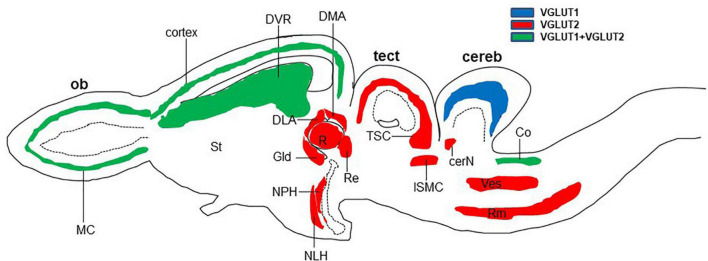
Schematic longitudinal section of turtle brain showing the glutamatergic neuron-expressing area by the expression of VGLUT1 (blue), VGLUT2 (red), or both (green). Glutamatergic neurons have been identified by the examination of vesicular glutamate transporter (VGLUT1 and VGLUT2) mRNA (Hussan, 2016; Sarkar and Atoji, 2018). cereb, cerebellum; cerN, cerebellar nucleus; Co, cochlear nucleus; DLA, anterior dorsolateral nucleus; DMA, anterior dorsomedial nucleus; DVR, dorsal ventricular ridge; Gld, dorsolateral geniculate nucleus; ISMC, magnocellular part of isthmic nucleus; MC, mitral cells; NLH, lateral hypothalamic nucleus; NPH, periventricular hypothalamic nucleus; ob, olfactory bulb; R, rotundal nucleus; Re, nucleus reuniens; Rm, medial reticular nucleus; St, striatum; tect, optic tectum; TSC, torus semicircular nucleus; Ves, superior vestibular nucleus.

**FIGURE 3:**
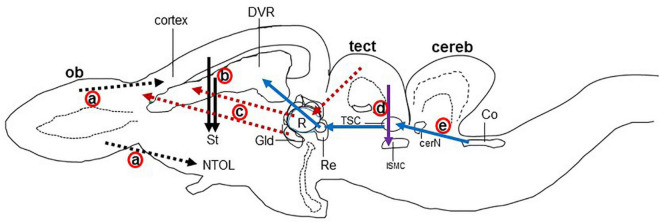
Schematic drawing of the longitudinal section through the turtle brain showing the major glutamatergic circuits. Circuits showing the (a) olfactory pathways (black dot arrow; Reiner and Karten, 1985; Lohman and Smeets, 1993), (b) corticostriatal pathways (black solid arrow; Desan, 1988; Hoogland and Vermeulen-vanderZee, 1989; Gonzalez et al., 1990), (c) visual pathways (red dot arrow; Kenigfest et al., 2007); (d) isthmo–tectal pathways (purple arrow; Schechter and Ulinski, 1979; Sereno and Ulinski, 1987), and (e) auditory pathways (blue arrow; Belekhova et al., 1985; Kunzle, 1986). cereb, cerebellum; cerN, cerebellar nucleus; Co, cochlear nucleus; DVR, dorsal ventricular ridge; Gld, dorsolateral geniculate nucleus; ISMC, magnocellular part of isthmic nucleus; MC, mitral cells; NTOL, nucleus of lateral olfactory tract; ob, olfactory bulb; St, striatum; R, rotundal nucleus; Re, nucleus reuniens; Rm, medial reticular nucleus; tect, optic tectum; TSC, torus semicircular nucleus; Ves, superior vestibular nucleus.

In reptiles, VGLUT1-3 genes have been identified in turtles and their mRNA distribution has been studied in the brains of turtles (Table 1; Hussan, 2016; Hussan et al., 2017; Sarkar and Atoji, 2018; Tosches et al., 2018), alligator (Briscoe and Ragsdale, 2018a), and lizard (Tosches et al., 2018; Norimoto et al., 2020). In turtles, VGLUT1 is strongly expressed in the olfactory bulb, the medial cortex, the dorsal cortex, the dorsomedial cortex, the lateral cortex, DVR, and the cerebellar cortex (Sarkar and Atoji, 2018). Conversely, VGLUT2 mRNA is weakly expressed in the telencephalon and strongly expressed in the thalamic and brainstem nuclei of turtles (Figure 2) (Hussan, 2016; Hussan et al., 2017; Sarkar and Atoji, 2018). Moreover, VGLUT3 mRNA is found only in the nucleus isthmi (parvocellular part), raphe nuclei, and cochlear nucleus of the turtle (Sarkar and Atoji, 2018). Among the anamniotes species of organisms, VGLUTs have been studied in the frog (Gleason et al., 2003), fish (Higashijima et al., 2004; Maruska et al., 2017), and Drosophila (Daniels et al., 2004). Moreover, in reptiles, although the investigation of GluRs has not yet been conducted extensively, some AMPA and NMDA receptors have been reported in the telencephalon and diencephalon of turtles by immunohistochemical methods (Fowler et al., 1999; Keifer and Carr, 2000). These results indicate the existence of neural circuits in the brain of the turtle that uses glutamate as its neurotransmitter. This review highlights the turtle as a unique model for the study of glutamatergic pathways and summarizes glutamatergic pathways in turtle brains, including the olfactory pathway, hippocampal–septal pathway, corticostriatal pathway, visual pathway, ascending auditory pathway, and brainstem–cerebellar pathway (Figure 3). In addition, attempts at comparative neuroanatomical studies of the neocortex will be presented.

## Glutamatergic circuits

**FIGURE 4:**
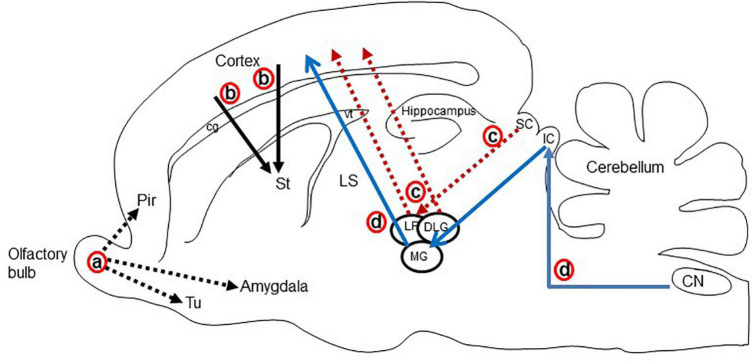
Schematic display of the major glutamatergic circuits in the longitudinal section through the rat brain. Circuits showing the (a) olfactory pathways (black dot arrow; Lledo et al., 2005; Ennis et al., 2015), (b) corticostriatal pathways (black solid arrow; Butler, 1994; Hintiryan et al., 2016), (c) visual pathways (red dot arrow; Butler and Hodos, 2005; Kenigfest et al., 2007), and (d) auditory pathways (blue arrow; Butler and Hodos, 2005; Ito and Oliver, 2010; Schofield et al., 2014). Cg, cingulum; CN, cochlear nucleus; DLG, dorsal lateral geniculate nucleus; IC, inferior colliculus; LP, lateral posterior thalamic nucleus; LS, lateral septal nucleus; MG, medial geniculate body; Pir, piriform cortex; SC, superior colliculus; Tu, olfactory tubercle; vt, ventricle.

**FIGURE 5:**
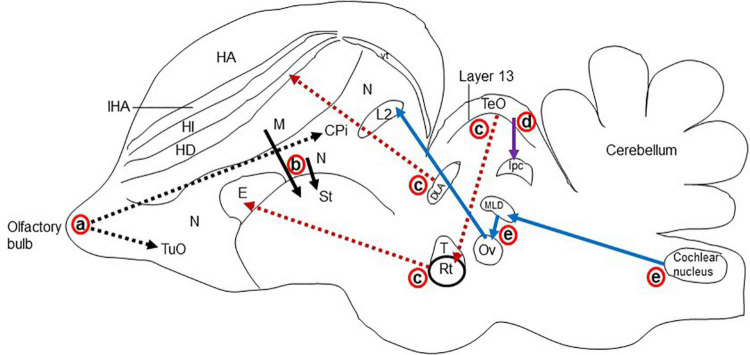
Schematic representation of the major glutamatergic circuits in the longitudinal section of the pigeon brain. Circuits showing the (a) olfactory pathways (black dot arrow; Reiner and Karten, 1985; Islam and Atoji, 2008), (b) corticostriatal pathways (black solid arrow; Wada et al., 2004; Islam and Atoji, 2008), (c) visual pathways (red dot arrow; Kenigfest et al., 2007; Atoji and Karim, 2014b); (d) isthmo–tectal pathways (purple arrow; Islam and Atoji, 2008; Karim et al., 2014), and (e) auditory pathways (blue arrow; Butler and Hodos, 2005; Atoji and Karim, 2014b). cpi, Piriform cortex; DLA, dorsolateral anterior nucleus; E, entopallium; HA, apical hyperpallium; HD, densocellular hyperpallium; HI, intercalated hyperpallium; IHA, interstitial apical hyperpallium; ISPC, parvocellular part of isthmic nucleus L2, field L2; M, mesopallium; MLD, lateral mesencephalic nucleus, dorsal part; N, nidopallium; Ov, ovoidal nucleus; Rt, rotundal nucleus; St, striatum; T, triangular nucleus; TeO, optic tectum; TuO, olfactory tubercle; vt, ventricle.

The major glutamatergic circuits of mammalian brains are found in the olfactory system, the cortex to striatum pathway, the hippocampus to septum pathway, the visual and auditory systems, and the cerebellum (Broman et al., 2004). Here, we provide information on the glutamate circuit in the turtle brain based on findings in the literature on VGLUTs and GluRs mainly by *in situ* hybridization or immunohistochemistry, and we also discuss the comparison of glutamate circuits in the brains of turtles (Figure 3), birds (Figure 4), and mammals (Figure 5).

### Olfactory pathway

There are main and accessory olfactory pathways in the olfactory system of amniotes except for humans, where the accessory olfactory system is absent (McGann, 2017). The accessory olfactory system is composed of the accessory olfactory bulb and the vomeronasal organ (Boehm, 2006). In the olfactory epithelium, specific neurons are present for olfaction, which receives the odor from the environment as chemical substances. The main olfactory bulb accepts projections from every olfactory receptor neuron (Lledo et al., 2005). In the olfactory pathway, olfactory receptor neurons send afferents to the glomerular layer and form synapses with the dendrites of mitral and tufted cells. These mitral and tufted cells further send afferent fibers to the olfactory cortex (Figure 4; Lledo et al., 2005; Ennis et al., 2015). In mammals, the olfactory pathway is suggested to be glutamatergic (Hisano et al., 2000; Kaneko et al., 2002). The main olfactory bulb is highly developed in reptiles, suggesting that the behavior of reptiles strongly depends on the olfactory system (Reiner and Karten, 1985; Lohman and Smeets, 1993). The projection neurons in the olfactory bulb of reptiles send their axons to the lateral cortex, hippocampus, olfactory tract, and amygdala (Figure 3; Reiner and Karten, 1985; Desan, 1988; Lohman and Smeets, 1993). The accessory olfactory bulbs in reptiles send projections to the posterior region of the central amygdaloid nucleus (Lohman and Smeets, 1993). In the olfactory bulb of turtles, mitral cells exhibited strong VGLUT1 and moderate VGLUT2 expression, identifying glutamate as the major neurotransmitter in the olfactory bulb afferent (Hussan, 2016; Hussan et al., 2017; Sarkar and Atoji, 2018). Alternatively, GluRs in the turtle olfactory bulb region and the central amygdaloid nucleus remain to be investigated. An electrophysiological study of the turtle olfactory bulb demonstrated that synaptic transmission from the axon terminal of an olfactory receptor neuron to mitral/tufted cell dendrites appeared as glutamatergic, and this synaptic transmission was regulated by NMDA and non-NMDA receptors (Berkowicz et al., 1994). Notably, in birds, mitral cells appeared as glutamatergic, and their target areas showed positive expression for GluRs (Figure 5; Wada et al., 2004; Islam and Atoji, 2008).

### Corticostriatal pathway

Projections from the dorsal pallium to the striatum appear to be a shared feature of amniotes (Butler, 1994). In mammals, the cerebral cortex provides a major input to the striatum (Hintiryan et al., 2016). Corticostriatal afferents arise from cortical pyramidal neurons and are located mostly in layer V, and to some extent in layers III and VI. These neurons utilize glutamate as their neurotransmitter and provide the major excitatory input to the striatum (Hisano et al., 2000). The corticostriatal afferent in mammals is topographically organized. Rostral areas of the striatum receive inputs from frontal areas and dorsolateral areas receive afferents from the sensorimotor cortex, while more caudal areas receive fibers from more rostral areas of the parietal cortex (Dudman and Gerfen, 2015; Hintiryan et al., 2016). In mammals, a strong expression of VGLUT1 mRNA is shown in layers V and VI of the cortex, while layers IV and VI show an intense expression for VGLUT2 mRNA (Ni et al., 1995; Fremeau et al., 2001). In contrast, GluA1-4 subtype mRNA and protein were labeled in the striatum (Keinanen et al., 1990; Martin et al., 1993; Sato et al., 1993). These findings indicate that corticostriatal pathways in mammals are glutamatergic (Figure 4). This speculation is also supported by electrophysiological and transcriptome studies in the neocortex of mammals (Andjelic et al., 2009; Tasic et al., 2018; Berg et al., 2021). In the telencephalic pallium of birds, VGLUT2 mRNA and protein were determined (Islam and Atoji, 2008; Atoji, 2011; Karim et al., 2014), and GluRs were expressed in the striatum (Ottiger et al., 1995; Wada et al., 2004; Islam and Atoji, 2008). In reptiles, corticostriatal projections have been found (Desan, 1988; Hoogland and Vermeulen-vanderZee, 1989; Gonzalez et al., 1990) and reported to be topographically organized (Desan, 1988; Gonzalez et al., 1990). Striatal projections originating from DVR have also been reported in reptiles (Gonzalez et al., 1990). In the turtle brain, VGLUT1 mRNAs have been found in pallial structures comprising the lateral cortex, dorsal cortex, medial cortex, DVR, and some amygdala nuclei (Sarkar and Atoji, 2018). Alternatively, the striatum showed positive expression for GluR immunoreactivity (Fowler et al., 1999). These existing data suggest that afferents to the striatum originating from the pallium in turtles are glutamatergic (Figure 3).

### Hippocampal–septal pathway

The medial cortex of reptiles is thought to be homologous to the hippocampal formation of mammals and birds, which is supported by embryological, hodological, and neurophysiological data (Atoji and Wild, 2004, 2006; Butler and Hodos, 2005; Jarvis, 2009; Tosches et al., 2018), although obvious morphological differences are present among the species. The medial area of the reptilian cortex has a substantial projection to the septum (Desan, 1988; Hoogland and Vermeulen-vanderZee, 1993). In the hippocampus of mammals, granule cells and pyramidal cells are glutamatergic (Fremeau et al., 2001). The pyramidal cells of CA3 of Ammon’s horn, which accept the mossy fiber from granule cells of the dentate gyrus, abundantly express GluA1-3 mRNAs (Sato et al., 1993). The lateral septal nucleus is strongly positive for GluA1-3 immunoreactivity, which accepts fibers from Ammon’s horn of the hippocampus (Martin et al., 1993). These data suggest that the hippocampal–septal pathway in mammals is glutamatergic. In birds, the hippocampal–septal pathway has been demonstrated (Atoji and Wild, 2004, 2006), suggesting that this pathway is glutamatergic (Islam and Atoji, 2008; Karim et al., 2014). In turtles, the medial cortical areas showed positive reactivity for glutamatergic neurons (Hussan et al., 2017; Sarkar and Atoji, 2018), which have strong projections to the septum, but GluRs have not yet been studied in the septum of turtles.

### Visual system

The visual stimulus from the retina reaches the cortex of the brain by the following two pathways in amniotes (Butler and Hodos, 2005): (1) Thalamofugal pathway: Here, the retina directly projects into the dorsolateral geniculate nucleus (Gld/dLGN) of mammals and reptiles or the anterior dorsolateral complex (DLA) of birds, from which the further projections enter into the dorsal lateral cortex of reptiles, avian Wulst, or the striate cortex, or the primary visual cortex of mammals (Kenigfest et al., 2007). (2) Tectofugal pathway: Here, the retina projects first to the optic tectum of birds and reptiles or the superior colliculus (SC) of mammals, from which projections enter into the nucleus rotundus (Rot) of reptiles and birds or to the lateral posterior nucleus and the pulvinar (Lp/pulv) in mammals; these projections, in turn, enter into the anterior DVR of reptiles or birds entopallium or mammalian extrastriate cortex and amygdala (Kenigfest et al., 2007; Day-Brown et al., 2010). In the thalamofugal pathway of mammals, Gld showed VGLUT2 mRNA expression (Hisano et al., 2002; Barroso-Chinea et al., 2007), and their target area, the striate cortex, demonstrated positive expression for GluA1-4 mRNA (Martin et al., 1993; Sato et al., 1993). In the tectofugal pathway of mammals, glutamatergic neurons are found in the SC and thalamic nuclei (Hisano et al., 2002; Kaneko et al., 2002; Barroso-Chinea et al., 2007). The thalamic nucleus and the cortical layers are the target areas for this visual pathway that are positive for GluA1-4 mRNA expression (Keinanen et al., 1990; Martin et al., 1993; Sato et al., 1993). Similar studies were conducted in birds demonstrating glutamatergic circuits in the ascending visual systems (Figure 5; Islam and Atoji, 2008; Atoji and Karim, 2014b; Karim et al., 2014). In the thalamofugal pathway of turtles (Hall et al., 1977; Balaban and Ulinski, 1981; Ulinski, 1986), glutamatergic neurons were found to be expressed in the Gld (Hussan, 2016; Hussan et al., 2017; Sarkar and Atoji, 2018), and GluR immunoreactivity was shown in the lateral dorsal cortex (Fowler et al., 1999). A pharmacological study showed that the thalamofugal visual pathway in turtles was glutamatergic, and this neurotransmission was mediated by NMDA and non-NMDA glutamate receptors (Larson-Prior et al., 1991). Furthermore, in the tectofugal pathway of turtles, the stratum griseum centrale (SGC) layer of the optic tectum receives projection from the retina, and the SGC further sends axons to the Rot. The Rot in turn projects to the anterior DVR (Hall et al., 1977; Bass and Northcutt, 1981). Vesicular glutamate transporter 2 mRNA was expressed in the SGC and Rot (Hussan, 2016; Hussan et al., 2017; Sarkar and Atoji, 2018), and immunoreactivity of GluRs was expressed in the Rot and anterior DVR (Fowler et al., 1999). These results suggested that both thalamofugal and tectofugal pathways in turtles are glutamatergic (Figure 3).

### Isthmo–tectal pathways

The nucleus isthmi are present within the midbrain and have visual responsiveness in all vertebrates. This nucleus can influence the visual process by direct modulation of isthmo–tectal pathways (Wang, 2003). This modulation is mediated by reciprocal connections between the nucleus isthmi and optic tectum (Sereno and Ulinski, 1987; Powers and Reiner, 1993). In turtles, two distinct nuclei are present in the isthmic complex, pars parvocellular (ISPC) and pars magnocellular (ISMC), which receive afferent fibers from the same side of the optic tectum (Sereno and Ulinski, 1987). The superficial layers of the optic tectum receive axons from the retinal ganglion cell (RGC) of the eye. These axons then form synapses with the dendrites of the stratum griseum periventricular (SGP) neurons (Schechter and Ulinski, 1979). Furthermore, these SGP neurons send axons to the ISPC and ISMC nuclei (Kunzle and Schnyder, 1984). In turtles, VGLUT2 mRNA was found to be expressed in the neurons of the SGP layer (Hussan, 2016; Hussan et al., 2017; Sarkar and Atoji, 2018), and GluR immunoreactivity was found in their projection targets ISMC and ISPC (Keifer and Carr, 2000). Thus, the turtle isthmo–tectal pathway is suggested to be glutamatergic (Figure 3).

### Ascending auditory pathways

The peripheral sound is conveyed through the middle tympanic ear to the basilar papilla, which then projects to the cochlear nuclei (Bruce, 2007). In turtles, the mesencephalic auditory center–torus semicircular nucleus receives signals from brainstem auditory nuclei, including cochlear nuclei and the superior olivary complex (Kunzle, 1986), and then relays information to the thalamic auditory center–nucleus reuniens (Belekhova et al., 1985). The nucleus reuniens projects further to the telencephalic auditory center, the ventromedial part of the anterior DVR (Balaban and Ulinski, 1981; Belekhova et al., 1985). In the auditory pathways of turtles, VGLUT2 mRNA was expressed in the cochlear nuclei, torus semicircular nucleus, and nucleus reuniens (Sarkar and Atoji, 2018). Further study is required to investigate glutamatergic neurons in the superior olivary nuclei and GluRs in all the projection nuclei of ascending auditory pathways. These findings suggested that the ascending auditory pathways in the turtle brain are glutamatergic (Figure 3). In birds, glutamatergic neurons have been demonstrated in all ascending auditory nuclei by the examination of VGLUT2 mRNA expression (Islam and Atoji, 2008; Karim et al., 2014). The projection nuclei of these auditory nuclei express GluR mRNA (Ottiger et al., 1995; Wada et al., 2004; Islam and Atoji, 2008; Karim et al., 2014). In pigeons, the thalamopallial auditory pathways were confirmed to be glutamatergic (Atoji and Karim, 2014b). In the mammalian auditory pathway, sound reaches the brainstem auditory nuclei and olivary and cochlear nuclei, then reaches the thalamic auditory nucleus and the medial geniculate nucleus, and finally sends it to the auditory cortex (Figure 4; Butler and Hodos, 2005; Schofield et al., 2014). Glutamatergic neuronal populations have been studied in ascending auditory pathways, where subcortical auditory nuclei mostly express VGLUT2 mRNA and the auditory cortex expresses VGLUT1 mRNA (Hackett et al., 2011; Ito et al., 2011). In rats, glutamatergic terminals were confirmed in the inferior colliculus received from lower auditory brainstem nuclei by retrograde labeling and by the examination of VGLUT1 and VGLUT2 mRNA expression (Ito and Oliver, 2010). In turtles, to confirm the ascending auditory pathways are glutamatergic, a tract–tracing method is required which has been used in mammals (Ito and Oliver, 2010) and birds (Atoji and Karim, 2014b).

### Brainstem–cerebellar pathways

In turtles, the cerebellum is an unbranched sheet consisting of the cerebellar cortex and the cerebellar nuclei (Nieuwenhuys et al., 1998). Compared to mammals, the cerebellum of reptiles possesses two principal afferent pathways: the mossy fiber–granule cell–parallel fiber pathway and the climbing fiber pathway. In efferent cerebellar connections, Purkinje cells send projections to the cerebellar and vestibular nuclei; cerebellar nuclei further send fibers to the vestibular nuclei and reticular nuclei (Nieuwenhuys et al., 1998). In the turtle, VGLUT1 mRNA was found to be highly expressed in the granular cells of the cerebellum (Sarkar and Atoji, 2018), which further send projections as parallel fibers to the Purkinje cells. The Purkinje cells showed positive expression for GluR immunoreactivity (Keifer and Carr, 2000). Again, the vestibular nuclei and reticular nuclei from which the mossy fiber originates appeared to be glutamatergic due to the expression of VGLUT2 mRNA (Hussan, 2016; Sarkar and Atoji, 2018). In contrast, the mossy fiber target is the granule cells that express GluRs (Keifer and Carr, 2000). Thus, these findings indicate that the parallel fibers and mossy fibers are glutamatergic. This notion is also supported by an electrophysiological study that showed that glutamatergic neurotransmission at the parallel fiber–Purkinje cell synapse is mainly regulated by AMPA receptors, whereas at the mossy fiber–granule cell synapse, it is regulated by AMPA and NMDA receptors (Larson-Prior et al., 1995). Notably, pontine nuclei have not been found in reptiles and may have separately evolved in bird and mammal brains (Butler and Hodos, 2005). In turtles, the inferior olivary nucleus was demonstrated morphologically to be the source of climbing fibers (Ariel, 2005), and an electrophysiological study suggested that the climbing fibers in turtles are excitatory (Chan et al., 1989). Together with electrophysiological data, the findings from the immunohistochemical study of GluRs in the Purkinje cells also suggest that the climbing fibers are glutamatergic. In mammals, the pontine nuclei and granule cells of the cerebellar cortex showed VGLUT1 mRNA expression, whereas VGLUT2 mRNA was expressed in inferior olivary and vestibular nuclei (Ni et al., 1995; Hisano et al., 2002; Hioki et al., 2003). Conversely, target areas of these glutamatergic nuclei found to be positive for GluR expression, such as GluA1-3 mRNAs, are expressed in the Purkinje cells, and GluA2 and GluA4 mRNAs are expressed in the granule cells (Keinanen et al., 1990). In pigeons, VGLUT2 mRNA appeared to be expressed in the granular layer of the cerebellum. Besides, VGLUT2 mRNA is expressed in the inferior olivary, pontine, and vestibular nuclei, from which the climbing and mossy fibers originate (Islam and Atoji, 2008). Purkinje cells showed positive expression for GluA1-3 mRNA, and granule cells exhibited expression for GluA1 and GluA2 mRNA in pigeons (Ottiger et al., 1995; Islam and Atoji, 2008). These observations suggest that similar glutamatergic circuits exist in the brainstem–cerebellar pathway of reptiles, birds, and mammals.

## Pallium

The telencephalon of the reptilian brain is composed of two main parts: the cerebral cortex and DVR. The cerebral cortex consists of three divisions, namely, the medial, dorsal, and lateral cortices (Butler and Hodos, 2005). The dorsal cortex of the turtle is thought to correspond with the reptilian forerunner of the mammalian neocortex (Butler and Hodos, 2005; Güntürkün et al., 2017). The dorsal cortex has several similar features to the mammalian neocortex, such as thalamic afferents from the dorsal lateral geniculate nucleus, cholinergic inputs from the striatum, noradrenergic afferents from the locus coeruleus, and serotonergic afferents from the raphe nucleus (Reiner, 1993). Similar to the mammalian striate cortex, the visual dorsal cortex of turtles also sends efferent fibers to the dorsal lateral geniculate nucleus of the thalamus, and the optic tectum (Hall et al., 1977; Ulinski, 1986). Furthermore, turtles have a somatosensory part in the rostral dorsal cortex that resembles that of mammals (Reiner, 1993; Aboitiz et al., 2002; Medina, 2007; Güntürkün et al., 2017; Tosches and Laurent, 2019), although the anterior DVR of reptiles also possesses sensory neuronal attributes such as somatosensory, visual, and auditory neurons (Manger et al., 2002). In turtles, the dorsal cortex is composed of three layers: (1) the molecular layer: a superficial and broad layer; (2) the cellular layer: the middle layer, thin, and cell body–rich; and (3) the subcellular layer: it is also thin and the most inner layer (Güntürkün et al., 2017). Two principal types of neurons are present in the dorsal cortex, pyramidal, and non-pyramidal interneurons. The pyramidal neurons are involved with the main input and output connections of the cortex, whereas the non-pyramidal neurons are local circuit GABAergic interneurons (Crockett et al., 2015; Güntürkün et al., 2017). Recent studies suggest that glutamatergic and GABAergic neurons were likely the main components of the pallium in all vertebrates (Suryanarayana et al., 2017; Spool et al., 2021).

**FIGURE 6:**
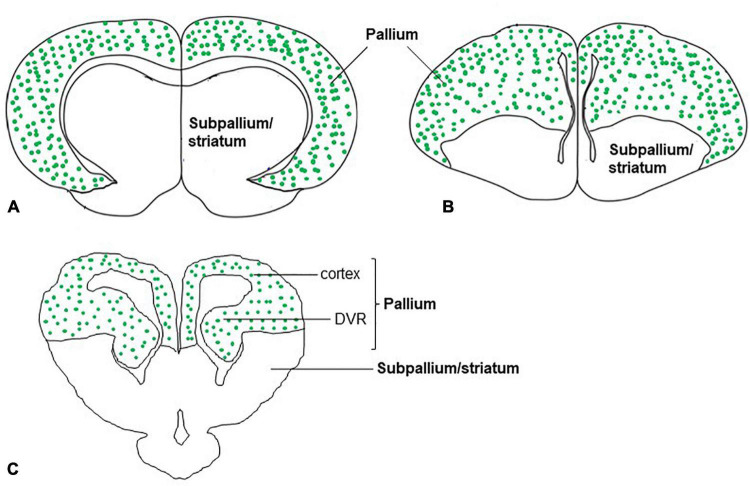
Schematic drawings of cross-sections through the mammal, bird, and, reptile (turtle) brains showing glutamatergic neurons (green dots) in the pallium of amniotes. The distribution of glutamatergic neurons in the pallium is based on the expression of **(A)** VGLUT1 and VGLUT2 mRNA in mammals (Ni et al., 1995; Fremeau et al., 2001), **(B)** VGLUT2 mRNA in birds (Islam and Atoji, 2008; Karim et al., 2014), and **(C)** VGLUT1 and VGLUT2 mRNA in reptiles (turtles) (Hussan, 2016; Sarkar and Atoji, 2018).

In the dorsal cortex of the turtle, pyramidal neurons showed that the expression of VGLUT1 mRNA (Sarkar and Atoji, 2018) was consistent with the immunohistochemical demonstration of glutamate (Fowler et al., 1999). In mammals, pyramidal neurons of the neocortex are highly positive for VGLUT1 mRNA (Ni et al., 1995). The medial region of the reptile cerebral cortex is comparable to the hippocampal formation of mammals, and the lateral cortex of reptiles is comparable to the piriform cortex of mammals (Tosches et al., 2018). In turtles, VGLUT1 mRNA is expressed in the medial and lateral cortices of the brain (Sarkar and Atoji, 2018; Tosches et al., 2018). Similarly, in the neocortex and hippocampal regions of adult rats, expression of VGLUT1 and VGLUT2 mRNA was observed (Ni et al., 1995; Fremeau et al., 2001). In addition to the cerebral cortex of turtles, the DVR appeared to show strong expression for VGLUT1 mRNA and weak expression for VGLUT2 mRNA. Recent research suggests that the evolution and features of neurons and circuits of DVR are unique to reptiles and birds (Tosches et al., 2018; Colquitt et al., 2021). Although molecular signature implicates that DVR cells originate from ventral pallium and develop under the control of a distinct set of transcription factors compared to the mammalian neocortex, DVR in reptiles and birds are functionally similar to the mammalian neocortex in terms of processing of sensory input. The anterior DVR has sensory connectivity resemblance to that of the mammalian ventral pallium (lateral amygdala) and possesses similar types of neurons which express VGLUT1 (Tosches et al., 2018; Colquitt et al., 2021). Finally, in birds, correspondingly to reptiles and mammals, pallial structures such as hyperpallium, mesopallium, nidopallium, arcopallium, dorsolateral corticoid area, temporo–parieto–occipital area, piriform cortex, hippocampal formation, posterior pallial amygdala, and nucleus teniae of the amygdala express VGLUT2 mRNA (Islam and Atoji, 2008; Karim et al., 2014). In summary, in the telencephalon of adult turtles, VGLUT1 and VGLUT2 mRNA expression, that is, distribution of glutamatergic neurons was found in a restricted region of the brain, the pallium (Figure 6). This finding is well agreed with that of mammals (Ni et al., 1995; Fremeau et al., 2001) and birds (Islam and Atoji, 2008; Karim et al., 2014) in terms of the pallial organization of glutamatergic neurons. Moreover, single-cell transcriptomics data of reptiles and birds also suggested the presence of conserved regions and cell types in the amniote pallium (Tosches et al., 2018; Colquitt et al., 2021). Further research would clarify whether these cell types in the turtles constitute a similar circuit structure to the mammalian neocortex as demonstrated in birds (Spool et al., 2021; Ditz et al., 2022).

## Functional involvement of glutamatergic neurons in the brains of turtles

In the brain of a turtle, glutamatergic neurons have been identified by examining the expression patterns of VGLUT1-3 mRNA. However, few data are available regarding the functions of glutamatergic neurons in the brains of turtles. The dorsomedial cortex of the reptile brain is assumed to be homologous to the hippocampal formation of mammals. Behavioral studies of turtles suggest that their dorsal and medial cortices are implicated in learning and memory (Lopez et al., 2003a,b). In the brain of a turtle, the medial cortex *in vitro* showed frequency potentiation, a type of synaptic plasticity regulated by AMPA receptors (Muñoz et al., 1998a). Moreover, long-term potentiation is induced by tetanic stimulation in the medial cortex of the turtle brain, which is also regulated by AMPA receptors (Muñoz et al., 1998b). The injection of MK-801 (NMDA receptor antagonist) and dorsal cortex lesions showed evidence for the participation of the dorsal cortex and NMDA receptor in the acquisition of a position habit of turtles (Avigan and Powers, 1995). The expression of VGLUT1 and 2 mRNA in the brains of turtles suggests that the dorsal and medial cortices have glutamatergic neurons (Hussan et al., 2017; Sarkar and Atoji, 2018). This morphological location of glutamatergic neurons in the dorsal and medial cortex of turtles is possibly involved in the learning and memory of turtles.

## The rationale for using the turtle as a model organism

To study the underlying pathways to protect neurons from ischemic conditions, the turtle has been considered an emerging model animal because turtles can survive in anoxia and postanoxic reoxygenation without any harm to the brain (Milton, 2019). Likewise, the turtle embryonic cortical cell culture neurons showed extreme resistance to glutamate exposures that are fatal to mammalian cortical neurons (Choi et al., 1987; Wilson and Kriegstein, 1991). In addition, the turtles’ phylogenetic position and morphological, developmental, and molecular features make them interesting to research (Wang et al., 2013; Naumann et al., 2015). The phylogenetic position of the turtle among amniotes has a big dispute. Morphological data align the turtle with lepidosaurs (tuatara, lizards, and snakes), whereas molecular and genomic data find the turtle should group with archosaurs (birds and crocodiles) (Hedges, 2012). The most peculiar morphological characteristic of the turtle is its shell composed of two parts a plastron (ventral) and a carapace (dorsal). There is no temporal opening in their compact skull (Rice et al., 2016). In recent years, the turtle model has been used to understand temperature-dependent sex determination, which is specific to many reptiles (Tezak et al., 2020; Merchant-Larios et al., 2021). Turtles are oviparous, and embryos can be hatched in the incubator. It is also easy to keep them in captivity (Cadi et al., 2004). The turtle is an excellent model to study developmental stages because its embryo is accessible for manipulation during its development (Tezak et al., 2020; Merchant-Larios et al., 2021). Moreover, as we have discussed in section 4, the dorsal cortex of turtles has several resemblances with the neocortex of mammals such as afferent connectivity including thalamic, adrenergic, serotonergic, cholinergic, and efferent connectivity to the thalamus and the brainstem (Reiner, 1993; Medina, 2007). The dorsal cortex of turtles and mammalian neocortex also share cell types and circuitry, although the turtle cortex has three layers instead of the mammalian six layers (Tosches et al., 2018). Thus, the study of turtle dorsal cortices is crucial to understanding the evolution of the mammalian neocortex (Reiner, 1993; Briscoe and Ragsdale, 2018b; Tosches and Laurent, 2019; Striedter and Northcutt, 2020). Furthermore, turtles are long-lived species with brain structures and functions similar to those of mammals and birds, particularly for learning and memory. Therefore, this species could be studied for many years, as they can survive for a long time and have long-term memory capacity (Congdon et al., 2003). The correlation between long lifespan and stress resistance has not yet been studied in turtles. However, the elevated levels of certain anti-stress proteins (heat shock protein-Hsp72), anti-apoptotic factors (Bcl-2), and antioxidant factors (FOXO3a) may make the turtle resistant to aging pathologies (Kesaraju et al., 2014; Reiterer and Milton, 2020; Reiterer et al., 2021).

## Glutamate in anoxia tolerance of turtle

The freshwater turtle *T. scripta* is a facultative anaerobe and can survive in anoxia for a day at room temperature and even a month at 3°C without brain damage. It can tolerate the shocking effect of re-oxygenation after the end of anoxia without affecting brain functions (Milton, 2019). In mammals, re-oxygenation is a great problem because ischemia-reperfusion frequently causes stroke or myocardial infarction (Naumann et al., 2015). In turtles, different stress-responsive mechanisms are activated in an anoxic environment such as preparing for hypometabolism, averting depolarization of the brain due to anoxia, upregulation of protective pathways, and downregulation of injurious or apoptotic pathways (Milton, 2019). Various factors are inherently present in the turtle which make them able to survive in the anoxic condition. For example, the turtle’s brain possesses a high level of glycogen which permits glycolysis for a longer period in anoxic conditions (Lutz et al., 2003; Packard and Packard, 2005). By contrast, lizards are reported to die in winter due to a lack of glycogen storage in the liver (Zani et al., 2012). In addition, delta-opioid receptors, whose activation is neuroprotective in both turtles and mammals (Pamenter and Buck, 2008; Tian et al., 2013; Subedi and Wang, 2020), are present four times more in the turtle cortex than in mammals (Xia and Haddad, 2001). Moreover, an elevated level of transcription regulator nuclear factor κB was observed in the liver of turtles and showed a beneficial role in anoxia tolerance (Krivoruchko and Storey, 2010). In fact, an *in vitro* study of a rat model suggested a neuroprotective role of NF-kB in brain ischemia by the activation of anti-apoptotic and suppression of pro-inflammatory gene expression (Simmons et al., 2016). Besides, an increased level of pituitary adenylate cyclase-activating polypeptide (PACAP) was observed in the brain of turtles in comparison to rats and human brains (Reglödi et al., 2001), which has a neuroprotective effect on glutamate-induced neurotoxicity and oxidative stress (Morio et al., 1996; Kaneko et al., 2018). A high level of heat shock protein such as Hsp72 was also reported in the turtle brain which causes neuroprotection through the modulation of reactive oxygen species (ROS) levels in anoxia/reoxygenation in turtles (Prentice et al., 2004; Kesaraju et al., 2014). Furthermore, the presence of abundant antioxidant activity such as methionine sulfoxide reductase and Forkhead box O3a (Foxo3a), a core regulator of the stress response, protects the turtle neurons during oxidative stress, particularly in anoxia and reoxygenation (Reiterer and Milton, 2020; Reiterer et al., 2021). Lastly, in the turtle brain, GABA is increased significantly (∼90-fold) during anoxia, which induces spike arrest by inhibiting the presynaptic release of glutamate and activating a postsynaptic inhibitory shunt (Nilsson and Lutz, 1991; Pamenter et al., 2011).

Turtle is the most anoxia tolerant among reptiles as well as vertebrates (Bickler and Buck, 2007). Meanwhile, all reptiles, or even all turtles, are not equally tolerant to anoxia. The soft-shelled turtle (*Apalone*) is an example of being relatively less anoxia tolerant having 50% mortality after 14 days of anoxia in water at 3°C (Reese et al., 2003). Among other members of reptiles, anoxia tolerance has been studied in some species of snakes (Seymour and Webster, 1975; Hermes-Lima and Storey, 1993; Rice et al., 1995). For example, garter snakes (*Thamnophis sirtalis parietalis*) are quite anoxia tolerant and can survive several hours without oxygen at 5°C (Hermes-Lima and Storey, 1993; Rice et al., 1995). Comparatively, very little data are available regarding the anoxia tolerance in lizards. Studies have shown that the European common lizard (*Lacerta vivipara*) tolerates the frozen state (3 days at −3°C) and utilizes its antioxidant system to cope with that hypoxic situation under freezing conditions (20 h, −2.5°C) (Costanzo et al., 1995; Voituron et al., 2006). In general, reptiles, amphibians, and fishes are more anoxia tolerant than mammals and birds (Bickler and Buck, 2007). However, some species of turtles such as *T. scripta* are extremely anoxia tolerant. Now, there is a question of whether these unique features of the turtles are due to the evolutionary divergence of reptiles or whether turtles have specific adaptations for anoxia tolerance. To resolve the issue, further extensive studies of anoxia tolerance and factors that are correlated with anoxia are required to investigate other species of reptiles such as lizards, snakes, and alligators from a phylogenetic point of view.

Controlling the excitatory neurotransmitter, particularly glutamate release and reuptake, is one of the crucial strategies of neuroprotection in anoxic turtles. In the mammalian brain, within a minute of anoxia, an increase in glutamate levels results in excitotoxic cell death which does not happen in the turtle brain. In the turtle brain, glutamate levels rather decrease and GABAergic signals increase (Hogg et al., 2014). In the early stage of anoxia (first hour), extracellular glutamate is maintained by decreasing glutamate release by neurons and reuptake continues by active glutamate transporters. At this stage, glutamate release is modulated by adenosine receptors and K^+^_*ATP*_ channels. In the later stage of anoxia, adenosine and GABA_*A*_ receptors regulate to maintain the low level of glutamate (Thompson et al., 2007). In addition to reducing extracellular glutamate release, postsynaptic GluRs activity is also reduced. For example, after 1 h of anoxia in the turtle dorsal cortex, a 65% decrease in NMDA receptor open probability (P_*open*_) has been observed (Buck and Bickler, 1998). Whole-cell AMPA receptor currents decline by 50–60% (Pamenter et al., 2008b; Zivkovic and Buck, 2010), whereas a 45–65% reduction in whole-cell NMDA receptor currents within 30 min of anoxia has also been reported (Pamenter et al., 2008a,c). During a short time (2 h) of anoxia of turtles, about a 35% rise in intracellular Ca^2+^ along with a decrease in the activity of NMDA receptors was observed (Bickler, 1998). The elevation of Ca^2+^ occurs due to its release from the mitochondria which regulate GluRs in anoxia (Pamenter et al., 2008c; Hawrysh and Buck, 2013; Hawrysh et al., 2022). These accumulated data suggest that glutamate is the key factor for anoxia resistance in the turtle brain and excitotoxic cell death in the mammalian brain. Inhibition of glutamate receptors or release of glutamate and enhancement of reuptake of glutamate could be the potential target to find therapeutics for the treatment of human diseases related to anoxia.

## Concluding remarks and the future directions

Turtles could be an invaluable research model to study human glutamate-related neurological disorders because of their extreme anoxia resistance based in part on the prominent glutamate-controlling system. In mammalian models of anoxia, both pathological and protective responses are induced. Conversely in turtles, the pathological pathways are largely suppressed and protective pathways are enhanced. Many of the mechanisms used by the turtle in anoxia are similar to those described as critical factors in mammalian ischemic/hypoxic preconditioned models (Milton, 2019). Thus, understanding the mechanism of anoxia tolerance, especially regarding the control of glutamate, in turtles may lead to finding a novel drug target to combat anoxia or hypoxia-related human diseases. For this purpose, first, it is necessary to establish glutamatergic circuits in the turtle brain, for which a prior expression study of glutamate-related factors is essential. In turtles, glutamatergic neurons have previously been identified by the expression of VGLUT1 and VGLUT2 mRNA (Hussan, 2016; Hussan et al., 2017; Sarkar and Atoji, 2018), but the protein expression studies of these VGLUTs remain to be explored. Full expression data of both mRNA and protein of GluRs is needed to study the turtle brain, although the protein expression of some GluRs has been studied in certain areas of the turtle brain (Fowler et al., 1999; Keifer and Carr, 2000). An ample amount of data on fiber connections of the turtle brain have previously been reported (Balaban and Ulinski, 1981; Ulinski, 1986; Reiner, 1993; Butler, 1994; Nieuwenhuys et al., 1998; Butler and Hodos, 2005; Chkheidze and Belekhova, 2005). Therefore, after completion of the gene expression study, the tract–tracing method is required to understand the glutamatergic circuits. In this review, we have shown possible glutamatergic circuits that could help to design further studies on the turtle brain. We have mentioned many benefits of the turtle as a research model; however, a few limitations also exist in studying turtle brains, including (1) the lack of a good brain atlas of the turtle brain, which might be due to the remarkable species variation among turtles; (2) the lack of genetically mutant turtle varieties (though it is possible to generate a transgenic turtle line using similar technology that is currently implemented in avian species) (Lee et al., 2020); and (3) the composition of turtle brains, such as their high glycogen (Lutz et al., 2003) and fiber content (Kalman et al., 1994) and large ventricles, which causes difficulty in the processing of turtle brain tissue for *in situ* hybridization or immunohistochemistry. Also, the application of genetic engineering for functional study in the turtle is still inadequate, although RNA interference (RNAi) methods have been implemented to knock down some genes in this species (Nayak et al., 2009; Kesaraju et al., 2014; Reiterer et al., 2021). These limitations could be overcome in the near future for further studies. The development of new technologies such as single-cell transcriptomics, neuroimaging, large-scale neural connectomics, optogenetics, and genetic engineering may become an advantage for comparative analysis of the brain including turtles. The turtle is not only important as an anoxic preconditioned model but also is interesting for understanding the evolution of the brain because it contains a brain structure that is in between mammals and birds such as the mammalian cortex and DVR in birds (Briscoe and Ragsdale, 2018b; Tosches and Laurent, 2019; Striedter and Northcutt, 2020).

In summary, in this review, we presented the expression areas of glutamate-related genes (VGLUTs and GluRs) and fiber connections of these areas in the turtle and compared them to those of birds and mammals. We also provided that glutamate is an important factor in anoxia tolerance of turtles and excitotoxic cell death in mammals. It is suggested that in mammals, CA1 neurons of the hippocampus are the most sensitive to hypoxia or ischemia (Michaelis, 2012; Shaw et al., 2021). Interestingly, the neurons in the turtle medial cortex, which is homologous to the hippocampus of mammals, possess epileptogenic properties suggesting their vulnerability (Shen and Kriegstein, 1986). Given that hippocampal cell types are well conserved in evolution (Tosches et al., 2018), the neurons of the turtle medial cortex would be therefore an interesting target for further investigation. Thus, with the glutamatergic circuits data, for instance, we would be able to determine which circuits are more likely to be affected by anoxia in the turtle brain by approaches such as pharmacology and electrophysiology and apply the findings to mammalian therapy. The integrated knowledge of the glutamatergic pathways shown in this review would act as a fundamental basis for advanced studies.

## Nomenclature

This review used the nomenclature for the forebrain and midbrain from the turtle brain atlas written by Powers and Reiner (1980) and for the rhombencephalon from the atlas written by Cruce and Nieuwenhuys (1974).

## Author contributions

MTH, AS, and HM wrote the manuscript. All authors contributed to the article and approved the submitted version.
